# *TRIM67* Promotes Non-Small Cell Lung Cancer Development by Positively Regulating the Notch Pathway through DLK1 Ubiquitination

**DOI:** 10.7150/jca.92723

**Published:** 2024-02-04

**Authors:** Jun Jiang, Xinjie Dong, Jixuan Liu, Ting Liu, Xiaoshuai Chen, Xiaolei Bian, Ming Li, Yafang Liu

**Affiliations:** Department of Pathology, Lequn Branch, The First Hospital of Jilin University, Changchun, China.

**Keywords:** DLK1, non-small cell lung cancer, Notch, *TRIM67*

## Abstract

Tripartite motif-containing 67 (TRIM67), a member of the TRIM protein family, is an E3 ubiquitin ligase. Our previous study revealed a relationship between TRIM67 expression and carcinogenesis, showing that TRIM67 expression is linked to p-TNM stage, lymph node metastasis, tumour size, cancer cell differentiation, and poor prognosis. Additionally, TRIM67 immunostaining results were associated with clinicopathological features. TRIM67 activated the Notch pathway in a favourable manner to enhance cell invasion, migration, and proliferation. Atypical ligand delta like non-canonical Notch ligand 1 (DLK1) inhibits the function of the Notch1 receptor, which in turn prevents activation of the Notch pathway. In addition, we investigated the mechanism by which TRIM67 influences the Notch pathway. We found that TRIM67 altered the behaviour of non-small cell lung cancer (NSCLC) cells by ubiquitinating DLK1 via its RING domain, which in turn activates the Notch pathway. Taken together, these findings indicate that TRIM67 may be involved in promoting the growth of NSCLC.

## Introduction

At present, lung cancer is one of the most prevalent malignant tumours worldwide, and the prognoses of early and advanced cases vary considerably 1-3. In advanced lung cancer, early detection and effective management of metastasis and recurrence are critical for reducing death rates. Thus, understanding the molecular processes underlying the initiation and spread of lung cancer and identifying new biomarkers are essential. They can improve the prognostic assessment of lung cancer and provide an experimental basis for the development of effective targeted therapy drugs and early therapies to improve patient survival rates.

Members of the tripartite motif-containing (TRIM) protein family play a role in numerous biological processes, including innate immunity, viral infection, and tumour emergence and progression. Their shared sequence, which consists of one or two B-box domains and a RING structure 4-9. TRIM67, a member of the TRIM family, functions as an E3 ubiquitin ligase; it mediates the stabilisation or degradation of target proteins through the attachment of ubiquitin molecules 10-12. The ubiquitin-proteasome proteolytic pathway plays a crucial role in regulating the abundance and activity of proteins, ultimately affecting cellular processes 13-15. By studying the function and regulatory pathways of TRIM67, researchers can explore the potential of TRIM67 as both a diagnostic biomarker and therapeutic target. Identifying specific molecular pathways and mechanisms in which TRIM67 is involved can contribute to the development of innovative treatment strategies, including the development of effective targeted therapy drugs. Furthermore, the discovery of additional TRIM proteins and their functions in lung cancer may provide further opportunities for therapeutic interventions. Identifying regulatory networks and interactions between TRIM proteins and other signalling molecules involved in lung cancer progression could lead to the development of novel combination therapies and personalised treatments.

The Notch pathway has been linked to decreased radiotherapy effects and chemotherapy resistance, in addition to its involvement in cell growth, proliferation, differentiation, and apoptosis 16. This signalling pathway has drawn considerable attention as a potential therapeutic target owing to its various effects on malignancies and tissue homeostasis 17-19. In recent years, the phenotypes of several malignancies, including breast cancer and T-cell leukaemia, have been linked to the Notch signalling system 20. There are four receptors (Notch1-Notch4) and five ligands (DLL-1, -3, and -4 and Jagged-1 and -2). The S2 location is made visible by a sequence of conformational changes in Notch caused by Notch4. Tumour necrosis factor-alpha-converting enzyme and metalloproteinase cleave the proteins, resulting in the formation of two fragments. The extracellular portion contains the N-terminally cleaved product, which ligand-expressing cells phagocytose. Gamma-secretase mostly cleaves the C-terminus at S3, releasing the active version of the Notch protein NICD 21. Cyclin D1, c-Myc, p21, p27, MMP9, RhoA, nuclear factor-kappa B, survivin, Slug, and Nanog are some of the downstream target genes of NICD, and the genes encoding these proteins meditate the onset and progression of cancer 22-24. Research has demonstrated that by controlling p53 stability, Notch1 can control the growth of lung cancer and prevent p53-regulated apoptosis.

We previously reported that TRIM67 positively regulates the Notch pathway to increase the migration, invasion, and proliferation of non-small cell lung cancer (NSCLC) cells 25. However, its exact mode of action remains unknown. The of this study was to elucidate the mode of action of TRIM67 in human NSCLC cells. TRIM67 was found to ubiquitinate delta like non-canonical Notch ligand 1 (DLK1) via its RING domain, which aided the activation of the Notch pathway. The findings of this study may have important implications for the development of novel therapeutic strategies for NSCLC. Targeting TRIM67 and the Notch pathway may offer a promising approach for the treatment of this lethal disease. In addition, the identification of the DLK1 substrate of TRIM67 provides valuable insights into the specific mechanisms by which this E3 ligase promotes Notch pathway signalling. Moreover, it is worthwhile to investigate the potential roles of TRIM67 and the Notch pathway in other types of cancer. Given the widespread involvement of the Notch pathway in cancer biology, TRIM67 may also play a role in other malignancies, such as breast cancer and leukaemia. Investigating the potential crosstalk between these pathways may provide new avenues for developing targeted cancer therapies. Furthermore, it is important to understand the regulation and expression of TRIM67 and its interaction with DLK1 in NSCLC and other cancers. Investigating the upstream regulators of TRIM67, such as microRNAs or transcription factors, may provide insights into the molecular mechanisms underlying its oncogenic role in cancer. Exploring the signalling pathways and molecular interactions that regulate the expression and function of TRIM67 may also reveal novel therapeutic targets for cancer treatment. Future studies aimed at unravelling the molecular details of this pathway and exploring its potential as a therapeutic target may have tremendous clinical significance and effect on cancer treatment.

## Materials and methods

### Cell culture

H1299 and A549 lung cancer cell lines were purchased from Zhong Qiao Xin Zhou Biotechnology (Shanghai, China). A549 cells were cultured in F12K medium (Zhong Qiao Xin Zhou Biotechnology) and H1299 cells were cultured in RPMI 1640 medium (Zhong Qiao Xin Zhou Biotechnology). Both media were supplemented with 10% foetal bovine serum (FB15015; Clark Biosciences, Richmond, VA, USA). Sterile culture flasks and plates (Thermo Fisher Scientific, Waltham, MA, USA) were used to culture the cells. The cell lines were cultured as described previously 26.

### Plasmid construction and transfection

Cell transfection was carried out using Lipofectamine 3000 reagent (Invitrogen, Carlsbad, CA, USA). For *TRIM67* knockdown, the cells were transfected for 48 h with control small-interfering RNA (siRNA; Shanghai Integrated Biotech Solutions, Shanghai, China) and *TRIM67*-specific siRNA. The cells were transfected with *TRIM67*-overexpression and RING finger domain-deletion mutant (ΔR) plasmids for 72 h. A matching empty pcDNA3.1 vector (Shanghai Integrated Biotech Solutions) was also used.

### Western blotting

Cellular proteins were extracted using lysis buffer (P0013; Beyotime Biosciences, Jiangsu, China) supplemented with a protease inhibitor cocktail (B14002; BioTool, Shanghai, China). Approximately 60 µg of each protein sample was separated via sodium dodecyl sulphate polyacrylamide gel electrophoresis on 10% gels. The separated proteins were subsequently transferred on to polyvinylidene fluoride membranes (Millipore, Billerica, MA, USA). After blocking for 2 h at 37°C in 5% skim milk (232100; Becton Dickenson, Franklin Lakes, NJ, USA), primary antibodies (Table 1) were applied to the membranes, which were then incubated at 4°C overnight. Subsequently, the membranes were washed and incubated with anti-mouse/rabbit IgG coupled with horseradish peroxidase (1:2000; Zsbg-bio, Beijing, China). Finally, a BioImaging system was used to determine immunoreactivity.

### Analyses of cell migration, invasion, and proliferation and colony formation

These assays were performed as described previously 25.

### Tumour formation in nude mice

The nude mice used in this study were handled according to the ethical guidelines for animal experiments of Jilin University. Prior to the experiments, 4-week-old female nude mice (obtained from Beijing Wekone Laboratory Animal Technology Co., Ltd., Beijing, China) were kept in a laminar flow cabinet under specific pathogen-free conditions for 1 week. Each mouse was subcutaneously inoculated with 1 × 10^7^ tumour cells (TRIM67-transfected A549 and H1299 cells or matching vector-transfected control cells) using 0.2 mL of sterile phosphate-buffered saline. The mice were euthanised 4 weeks after the injection and tumour growth was observed. The protocols for animal care and experiments were approved by the Jilin University Animal Ethics Committee.

### Coimmunoprecipitation assays

The cells were lysed in a 10-cm Petri dish using NP40. The resulting lysate was centrifuged at 5000 × *g* for 15 min and then mixed with 60 μL of Protein A/G Sepharose, blocked for 2 h, and centrifuged at 1000 rpm to extract magnetic beads. The remaining protein lysate was evenly divided into two parts, which were then treated with 5 μg of target antibody and anti-mouse/rabbit IgG. Subsequently, the samples were shaken and stored in a 4°C chromatographic cabinet overnight. The next day, 25 μL of agarose A/G magnetic beads was added to each tube, and the samples were incubated at 4°C for 6 h. The samples were then washed with lysis buffer, the cell lysis solution was boiled in water for 10 min, and western blotting was performed to examine the samples.

### Ubiquitination assay and immunoprecipitation

A549 and H1299 cells were transfected with FLAG-tagged TRIM67 and si-TRIM67 together with HA-ubiquitin (Ub) and then treated with the 26S proteasome inhibitor MG132. The cells were also transfected with Ub and Flag-tagged wild-type or ΔR of TRIM67. Subsequently, immunoprecipitation with an anti-DLK1 antibody and anti-HA immunoblotting were performed, and the degree of DLK1 ubiquitination was assessed.

### Statistical analysis

Statistical analyses were performed using SPSS (version 17.0; SPSS Inc., Chicago, IL, USA). Student's *t*-test was used to examine statistically significant variations in TRIM67 expression and clinical characteristics as described previously 27. Results with *P* < 0.05 were considered statistically significant.

## Results

### TRIM67 induces Notch signalling through DLK1 in NSCLC cells

**Figure 1:**
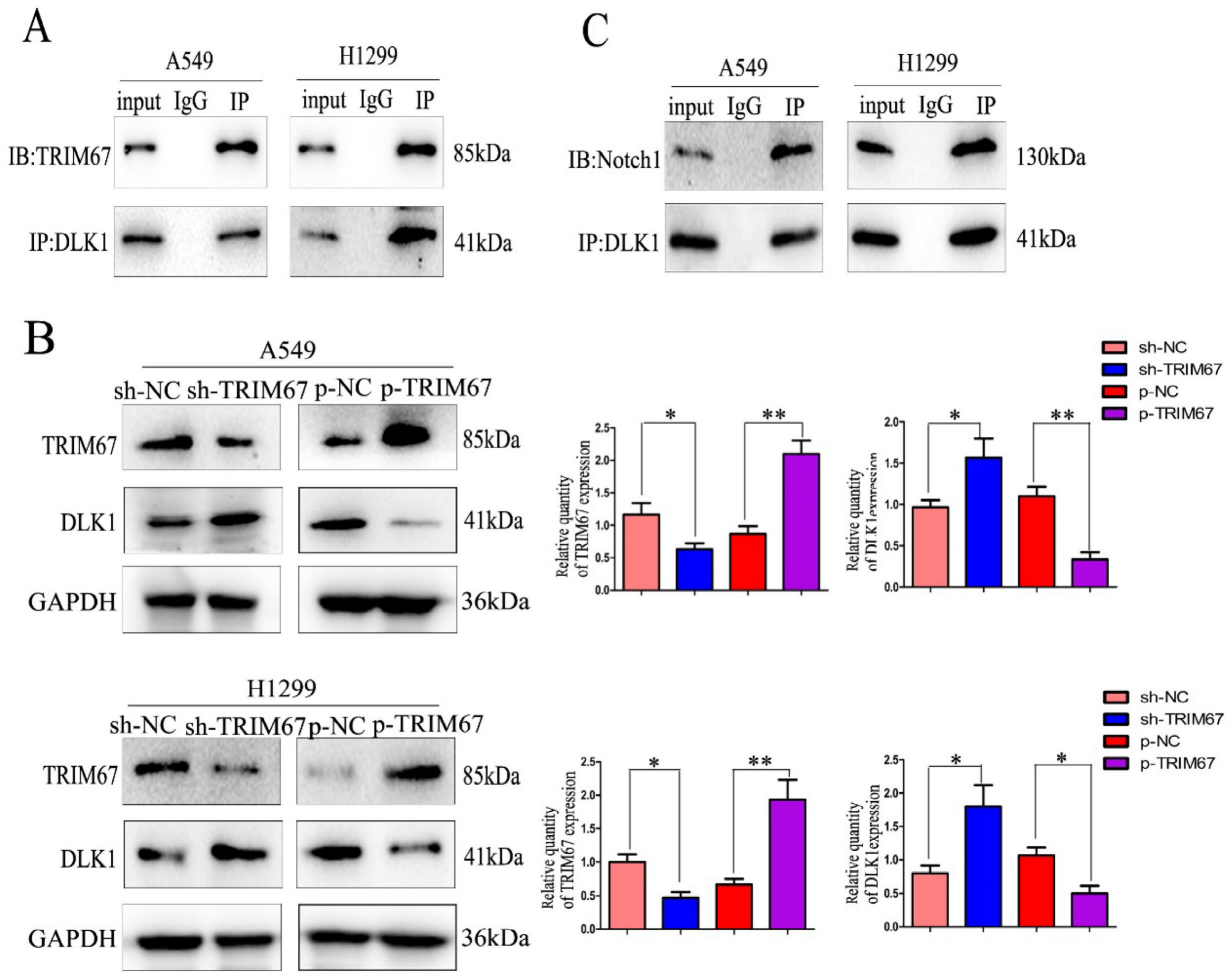

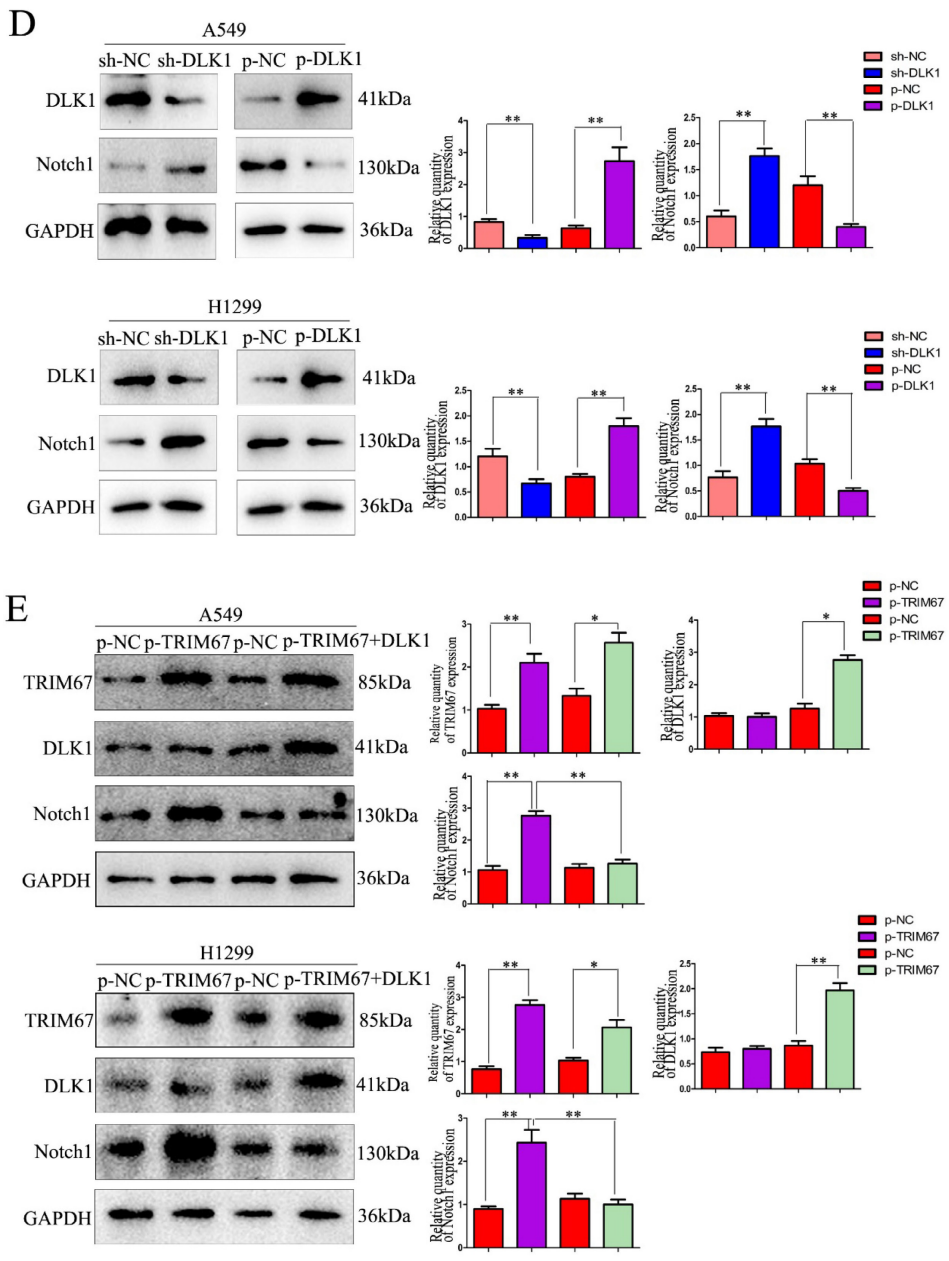
TRIM67 interacts with DLK1. (**A**) Interactions between TRIM67 and DLK1 in A549 and H1299 cells determined using coimmunoprecipitation. (**B**) Effects of the levels of TRIM67 on the expression of DLK1 in A549 and H1299 cells. Relative quantification analysis was based on grayscale values. *P < 0.05, **P < 0.01, vs. control cells. (**C**) Interactions between DLK1 and Notch1 in A549 and H1299 cells determined using coimmunoprecipitation. (**D**) Effects of the levels of DLK1 on the expression of Notch1 in A549 and H1299 cells. Relative quantification analysis was based on grayscale values. *P < 0.05, **P < 0.01, vs. control cells. (**E**) Changes in the level of Notch1 after transfection of A549 and H1299 cells with DLK1 and TRIM67. Relative quantification analysis was based on grayscale values. *P < 0.05, **P < 0.01, vs. untransfected cells. TRIM67, tripartite motif-containing 67; DLK1, delta like non-canonical Notch ligand 1.

Drawing on previous study findings 25, we investigated the effect of TRIM67 on the Notch signalling system, focusing on DLK1 expression. DLK1 belongs to the family of Notch EGF-like receptors and ligands; it controls various biological processes, such as transcription, transduction, and protein transport and degradation. Additionally, it has been associated with the development and spread of a number of cancers 28-30. Using coimmunoprecipitation, we confirmed the association between TRIM67 and DLK1 (Fig. 1A). We altered the expression of TRIM67 in A549 and H1299 cells to better understand its relationship with DLK1. *TRIM67* knockdown upregulated the expression of DLK1, whereas its overexpression had the reverse effect (Fig. 1B). According to reports, DLK1 is a peculiar ligand with the ability to block the receptor function of Notch1, which blocks the activation of the Notch pathway 31,32.

Here, coimmunoprecipitation confirmed the relationship between DLK1 and Notch1 (Fig. 1C). Subsequently, we altered the expression of DLK1 in A549 and H1299 cells. Downregulation of DLK1 expression increased Notch1 expression, whereas its overexpression had the opposite effect (Fig. 1D). A549 and H1299 cells were simultaneously transfected with plasmids containing *TRIM67* and *DLK1*, and the results showed that DLK1 reduced the ability of TRIM67 to promote Notch1 expression (Fig. 1E). These results indicate that DLK1 is involved in the mechanism by which TRIM67 controls the Notch pathway.

### TRIM67 is involved in the ubiquitin-dependent degradation of DLK1

**Figure 2:**
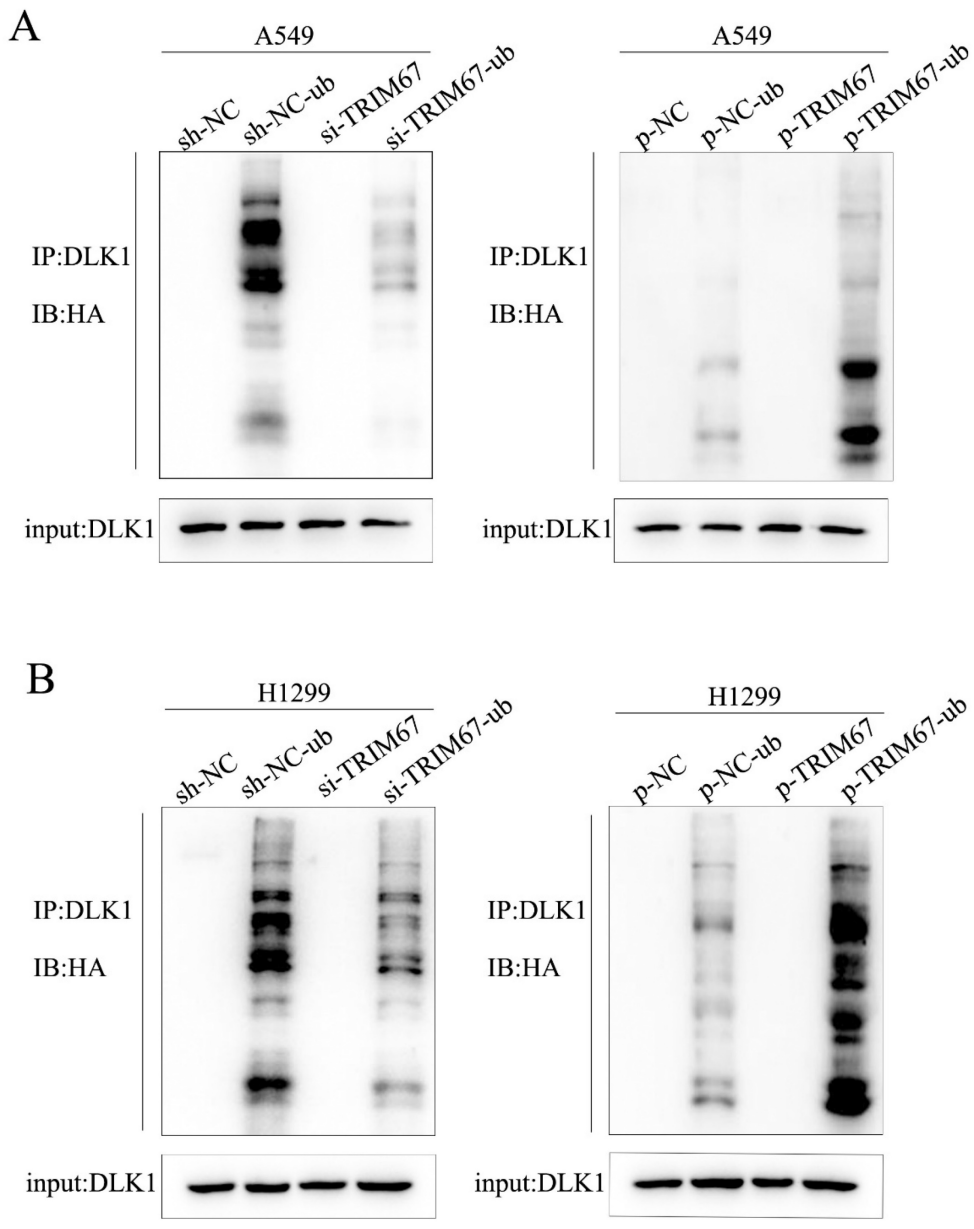
Effects of TRIM67 expression on DLK1 ubiquitination. (**A**) A549 cells transfected with si-TRIM67 or TRIM67-overexpressing plasmid along with HA-ubiquitin (Ub). The levels of DLK1 ubiquitination were evaluated using immunoprecipitation with the anti-DLK1 antibody, followed by anti-HA immunoblotting. (**B**) The same analysis was performed in H1299 cells. TRIM67, tripartite motif-containing 67; DLK1, delta like non-canonical Notch ligand 1.

We investigated how TRIM67 and DLK1 work together to activate the Notch signalling pathway. TRIM67 is active as an E3 ubiquitin ligase. Thus, we postulated that TRIM67 promotes DLK1 ubiquitination, which facilitates the activation of the Notch pathway. In A549 cells, we observed a significant correlation between the level of DLK1 ubiquitination and TRIM67 expression (Fig. 2A), and similar outcomes were observed in H1299 cells (Fig. 2B). These results indicate that TRIM67 is involved in the ubiquitin-dependent degradation of DLK1, which activates the Notch pathway.

### TRIM67 is involved in ubiquitin-dependent DLK1 degradation through its RING domain

**Figure 3:**
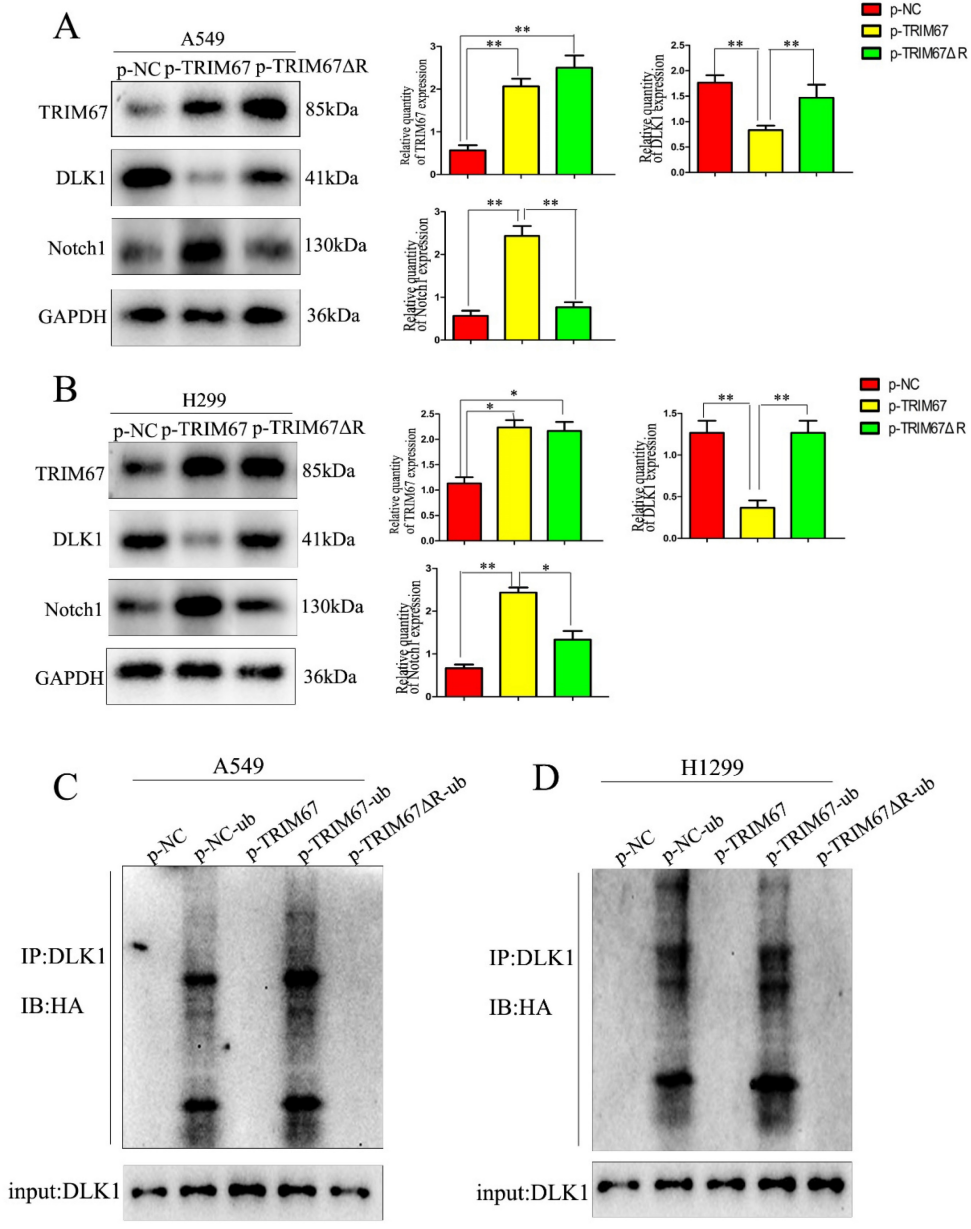
TRIM67 is involved in DLK1 ubiquitination and degradation through its RING finger domain, thereby regulating the Notch pathway. (**A**) A549 cells were transfected with either a wild-type TRIM67 plasmid or a plasmid carrying a TRIM67 RING finger domain-deletion mutant (TRIM67 ΔR). The expression of DLK1 and Notch1 was analysed using western blotting. Relative quantification analysis was based on grayscale values. *P < 0.05, **P < 0.01, vs. control cells. (**B**) The same analysis was performed in H1299 cells. (**C**) A549 cells were transfected with either a wild-type or TRIM67 ΔR plasmid. Immunoblotting was performed to determine the effects of RING-deleted TRIM67 on the levels of DLK1. Flag-tagged wild-type or TRIM67 ΔR was expressed in cells along with Ub. The levels of DLK1 ubiquitination were evaluated using immunoprecipitation of DLK1 with anti-DLK1 antibodies followed by anti-HA immunoblotting. (**D**) The same analysis was performed in H1299 cells. TRIM67, tripartite motif-containing 67; DLK1, delta like non-canonical Notch ligand 1; Ub, HA-ubiquitin.

We investigated whether the TRIM67 domain is responsible for ubiquitination. From a literature survey, we found that RING is the major domain of E3 ubiquitin ligases 33. Thus, we used a plasmid containing *TRIM67* with RING domain deletion (TRIM67 ΔR) to transfect cells. We found that TRIM67 ΔR reversed the effects on DLK1, but the Notch1 and DLK1 levels remained unchanged (Fig. 3A and B). This finding suggested that the RING domain of TRIM67 is crucial for the ubiquitination of DLK1. Experiments with ubiquitination further confirmed this supposition. We found that TRIM67 ΔR had no effect on DLK1 ubiquitination levels (Fig. 3C). Based on these findings, TRIM67 plays a role in the ubiquitin-dependent degradation of DLK1 through its RING domain, which in turn triggers the Notch pathway. Similar results were observed in H1299 cells (Fig. 3D).

### TRIM67 regulates cell migration, invasion, and proliferation through the RING domain

**Figure 4:**
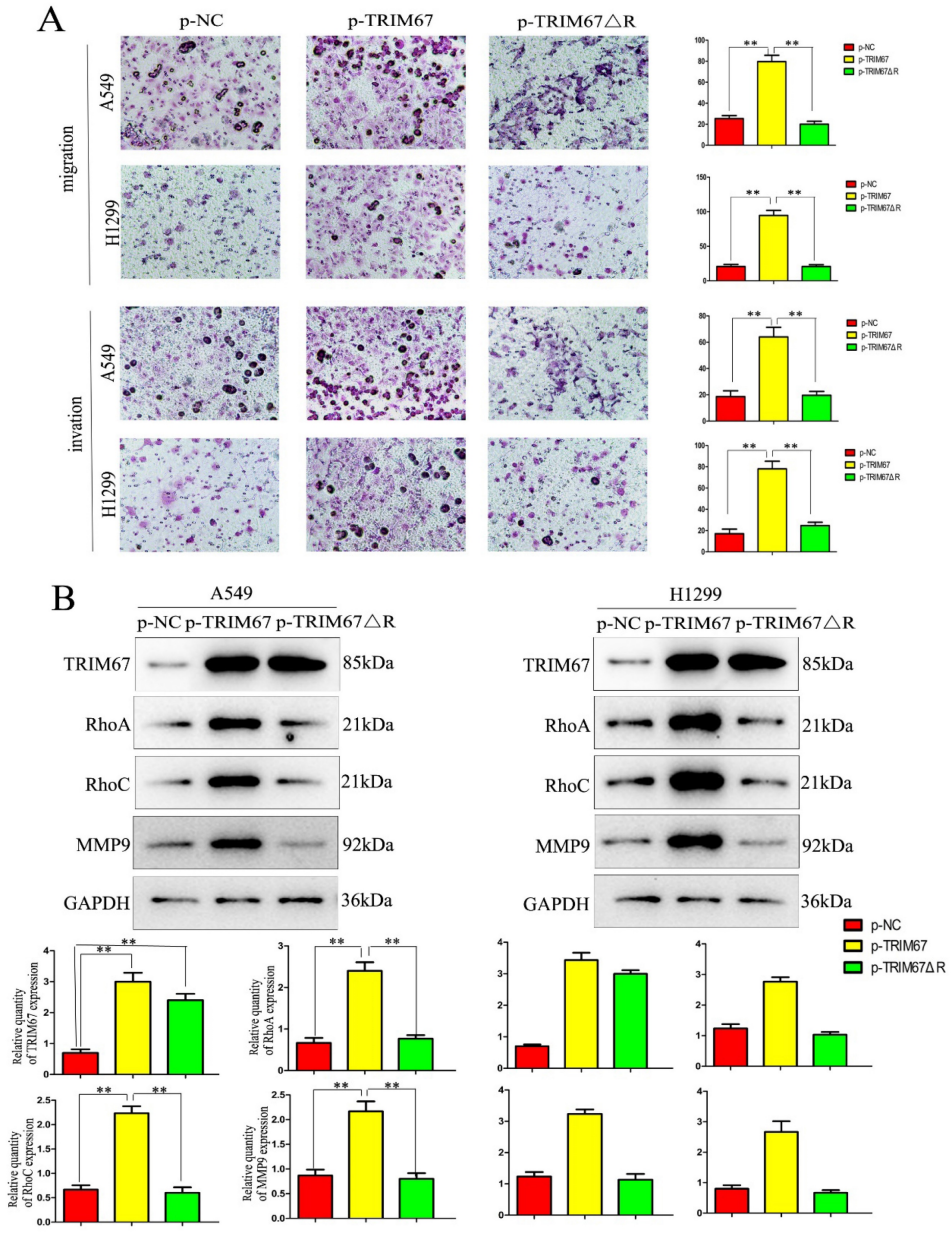

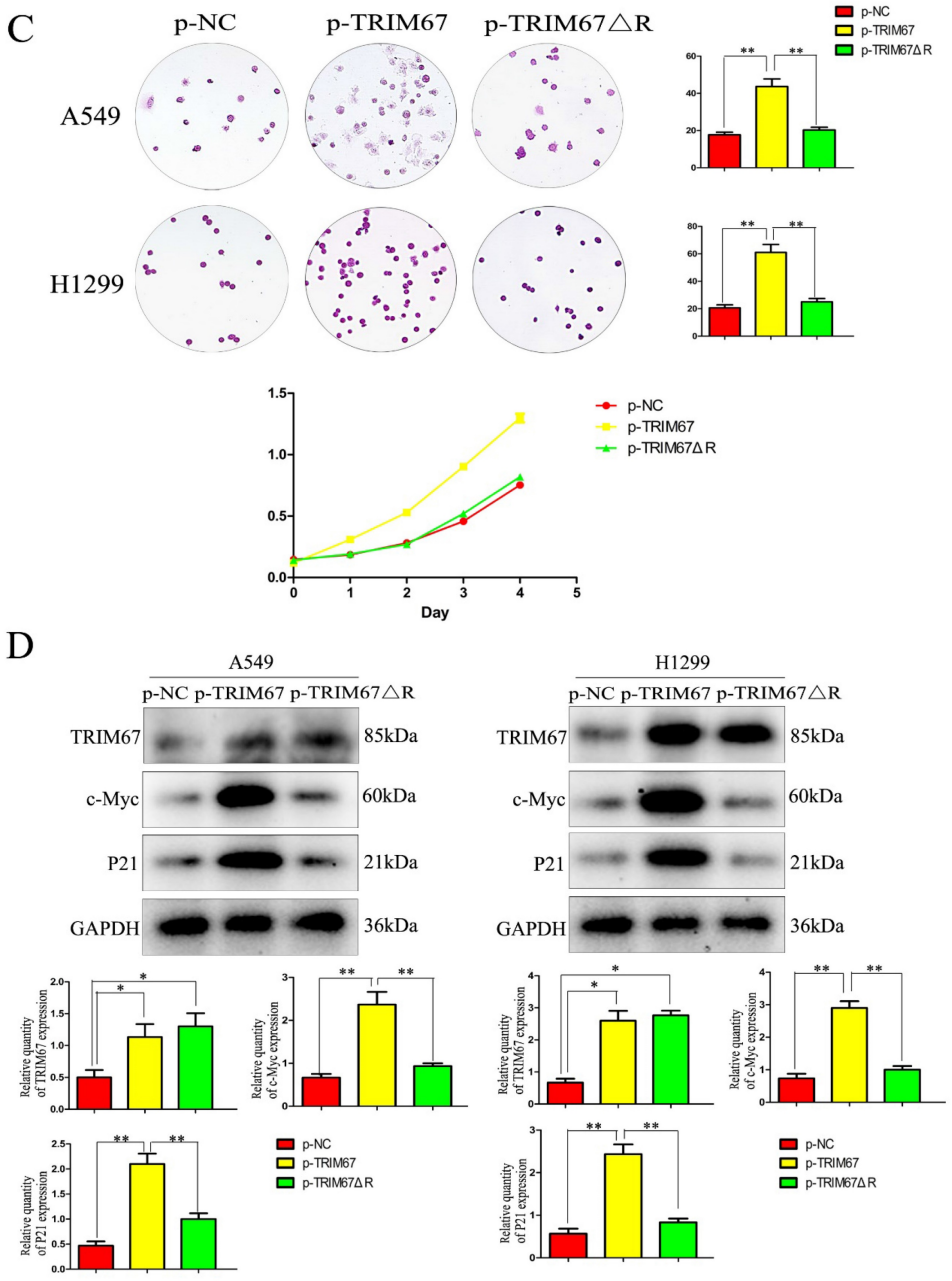
Effects of deleting the RING domain of TRIM67 on cell migration, invasion, and proliferation. (**A**) Transwell assays were performed to assess cell migration and invasion in the context of either overexpressing TRIM67 or expressing a RING domain deletion of TRIM67. *P < 0.05, **P < 0.01, vs. control cells. (**B**) A549 and H1299 cells were transfected with either a wild-type or TRIM67 ΔR plasmid. The expression of proteins associated with cell migration and invasion in transfected cells was investigated. Relative quantification analysis was based on grayscale values. *P < 0.05, **P < 0.01, vs. control cells. (**C**) Colony formation assays and cell counting kit-8 assays were performed to assess cell proliferation after transfection with either of the two plasmids. *P < 0.05, **P < 0.01, vs. control cells. (**D**) The effects of deleting the RING domain of TRIM67 on the expression of proteins associated with cell proliferation in A549 and H1299 cells. Relative quantification analysis was based on grey scale values. *P< 0.05, **P< 0.01, vs. control cells. TRIM67, tripartite motif-containing 67.

Next, we examined how TRIM67 ΔR affected cell invasion, migration, and proliferation. We transfected A549 and H1299 cells with TRIM67 ΔR or wild-type TRIM67 plasmids. We found that loss of the RING domain of TRIM67 diminished its ability to promote cell invasion, migration, and proliferation (Fig. 4A and B). Furthermore, its upregulating effect on associated protein levels were similarly diminished (Fig. 4C and D). These findings demonstrate the importance of the RING domain of TRIM67.

### TRIM67 promotes cell proliferation *in vivo*

**Figure 5:**
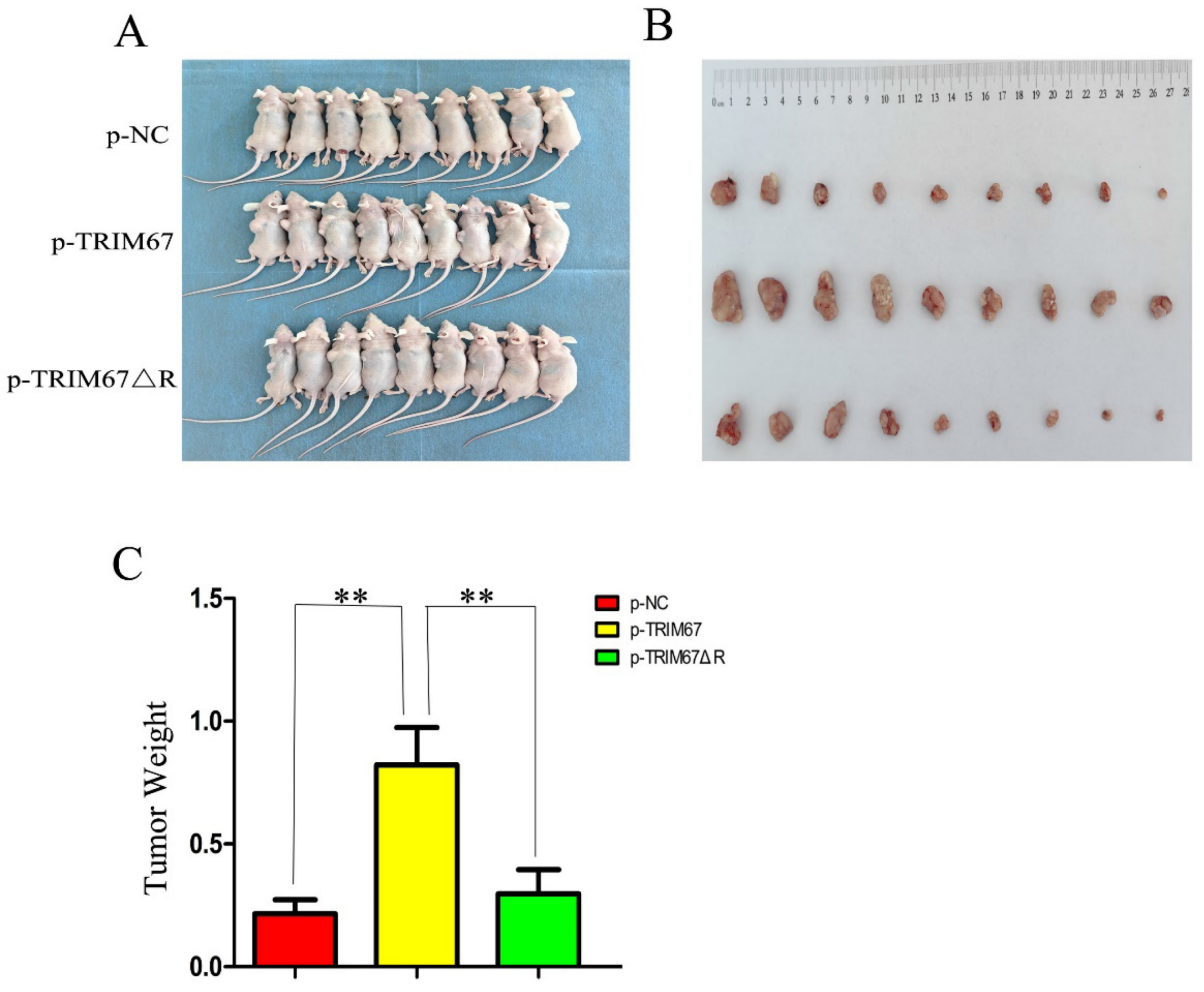
Effect of wild-type and TRIM67 ΔR on the volume of xenografted tumours *in vivo.* (**A**) Comparison of gross photos of nude mice. (**B**) Gross picture of subcutaneous tumour. (**C**) Histograms of subcutaneous tumorigenesis.

Using G418 and pertinent plasmids, we produced A549 cell lines that were stably transfected. We found that xenograft tumour weight and volume were positively affected by variations in TRIM67 expression in these cells. In contrast, this effect was diminished following the ablation of the RING domain (Fig. 5A-C). These *in vivo* findings provide additional evidence that TRIM67 overexpression promotes NSCLC cell proliferation, with the RING domain being a crucial component in this process.

## Discussion

TRIM67 is a member of the TRIM family and is found in the nucleus and cytoplasm. Here, we discovered that TRIM67 influences the malignant behaviour of tumour cells by promoting the proliferation, migration, and invasion of NSCLC cells via the Notch pathway and the expression of downstream proteins, including RhoA, RhoC, MMP-9, P21, c-Myc, and epithelial-mesenchymal transition-related markers 25. However, the processes underlying these roles are unknown.

In this study, we investigated the function of TRIM67 in the Notch signalling cascade. We used coimmunoprecipitation to validate the interaction between TRIM67 and DLK1, targeting DLK1 according to previous reports 31. DLK1 expression and TRIM67 expression were found to be negatively linked, although the fundamental process is still unknown. We hypothesised that the E3 ubiquitin ligase TRIM67 influences DLK1 levels via its ubiquitination, and this was validated using our ubiquitination experiment. The RING domain is recognised as the principal functional domain of ubiquitin ligases 33. We performed transfection with TRIM67 ΔR mutants to verify whether TRIM67 regulates Notch signalling via the ubiquitination of DLK1 through its RING domain. These results were also validated *in vivo*.

Taken together, our findings suggest that DLK1 is ubiquitinated by TRIM67 through its RING domain, controlling the Notch system, and that it holds potential as a prognostic biomarker and therapeutic target for NSCLC. However, it is not clear which TRIM67 binding site is involved in this process, and further investigation is needed to determine the exact mechanism(s) through which DLK1 influences the Notch pathway. Nonetheless, our research provides important new insights into the roles and modes of action of TRIM67 in carcinogenesis.

## Figures and Tables

**Table 1:** List of antibodies used in western blotting in this study

Antibody name	Source	Catalogue number	Host	Dilution
TRIM67	Sigma-Aldrich	HPA034776	Rabbit	1:50
TRIM67	Sigma-Aldrich	SAB2103188	Rabbit	1:100
GAPDH	Beyotime	AF0006	Mouse	1:1000
RhoA	Cell Signaling Technology Inc.	2117	Rabbit	1:500
RhoC	Cell Signaling Technology Inc.	3430	Rabbit	1:500
MMP9	Cell Signaling Technology Inc.	13667	Rabbit	1:500
P21	Cell Signaling Technology Inc.	2947	Rabbit	1:1000
c-Myc	Cell Signaling Technology Inc.	13987	Rabbit	1:1000
DLK1	Cell Signaling Technology Inc.	2069	Rabbit	1:500
HA	TransGen Biotech	HT301	Mouse	1:1000
Notch1	Cell Signaling Technology Inc.	3608	Rabbit	1:500

## Abbreviations

DLK1: delta like non-canonical Notch ligand 1
NSCLC: non-small cell lung cancer
siRNA: small-interfering RNA
TRIM67: tripartite motif-containing 67
Ub: ubiquitin
